# Effect of the third dose of BNT162b2 vaccine on quantitative SARS-CoV-2 spike 1–2 IgG antibody titers in healthcare personnel

**DOI:** 10.1371/journal.pone.0263942

**Published:** 2022-03-02

**Authors:** Maria Elena Romero-Ibarguengoitia, Diego Rivera-Salinas, Yodira Guadalupe Hernández-Ruíz, Ana Gabriela Armendariz-Vázquez, Arnulfo González-Cantú, Irene Antonieta Barco-Flores, Rosalinda González-Facio, Laura Patricia Montelongo-Cruz, Gerardo Francisco Del Rio-Parra, Mauricio René Garza-Herrera, Jessica Andrea Leal-Meléndez, Miguel Ángel Sanz-Sánchez

**Affiliations:** 1 Department of Investigation, Hospital Clínica Nova de Monterrey, San Nicolás de los Garza, Nuevo León, Mexico; 2 Vice-Presidency of Academic Affairs in Health Sciences, School of Medicine, Universidad de Monterrey, San Pedro Garza García, Nuevo León, Mexico; University of Hail, SAUDI ARABIA

## Abstract

**Background:**

Vaccination is our main strategy to control SARS-CoV-2 infection. Given the decrease in quantitative SARS-CoV-2 spike 1–2 IgG antibody titers three months after the second BNT162b2 dose, healthcare workers received a third booster six months after completing the original protocol. This study aimed to analyze the quantitative SARS-CoV-2 spike 1–2 IgG antibody titers and the safety of the third dose.

**Material and methods:**

A prospective longitudinal cohort study included healthcare workers who received a third booster six months after completing the BNT162b2 regimen. We assessed the quantitative SARS-CoV-2 spike 1–2 IgG antibody titers 21–28 days after the first and second dose, three months after the completed protocol, 1–7 days following the third dose, and 21–28 days after booster administration.

**Results:**

The cohort comprised 168 participants aged 41(10) years old, 67% of whom were female. The third dose was associated with an increase in quantitative antibody titers, regardless of previous SARS-CoV-2 history. In cases with a negative SARS-CoV-2 history, the median (IQR) antibody titer values increased from 379 (645.4) to 2960 (2010) AU/ml, whereas in cases with a positive SARS-CoV-2 history, from 590 (1262) to 3090 (2080) AU/ml (p<0.001). The third dose caused a lower number of total (local and systemic) adverse events following immunization (AEFI) compared with the first two vaccines. However, in terms of specific symptoms such as fatigue, myalgia, arthralgia, fever, and adenopathy, the proportion was higher in comparison with the first and second doses (p<0.05). The most common AEFI after the third BNT162b2 vaccine was pain at the injection site (n = 82, 84.5%), followed by fatigue (n = 45, 46.4%) of mild severity (n = 36, 37.1%).

**Conclusion:**

The third dose applied six months after the original BNT162b2 regimen increased the quantitative SARS-CoV-2 spike 1–2 IgG antibody titers. The booster dose was well tolerated and caused no severe AEFI.

## Introduction

In the first trimester of 2020, The World Health Organization (WHO) recognized the spread of SARS-CoV-2 as a pandemic [1]. Its negative and severe impact on society, the economy, and health due to its significant morbidity and mortality prioritized vaccine development to control the disease worldwide. Many vaccines were developed for emergency use [2]. The vaccines could lead to the development of spike-specific IgG antibodies against SARS-CoV-2, so serology assays have been used to detect the spike protein domain antibodies induced by vaccination or prior viral exposure [3].

The vaccine type depends on its mechanism of action. To date, the different SARS-CoV-2 vaccines designs are: mRNA (BioNTech-Pfizer/BNT162b2, Moderna/mRNA-1273), adenovirus viral vector (Oxford-AstraZeneca/ChAdOx1, Gam-COVID-VAC/Sputnik V, Ad26.COV2.S/Jannsen, CanSinoBio/Ad5-nCoV), protein subunit (Novavax/NVX-CoV2373, Medicago CoVLP), whole-cell inactivated virus vaccines (Inova/CoronaVac, Sinopharm/ BBIBP-CorV), and DNA vaccines (INO-4800 and ZyCoV-D) [4].

The Pfizer and BioNTech vaccine, hereafter referred to as BNT126b2, was the first SARS-CoV-2 vaccine to show promising efficacy. On November 18, 2020, it was proven to be 95% effective against symptomatic and severe disease [5]. As a result, in December 2020, the WHO and the Food and Drug Administration (FDA) authorized this vaccine for emergency use, the first SARS-CoV-2 vaccine to receive emergency approval [6, 7].

The Centers for Disease Control and Prevention (CDC) and the U.S. Food and Drug Administration (FDA) have authorized a third BNT162b2 vaccine to the following individuals: 1) individuals > 50 years of age with a medical condition; 2) individuals > 18 years of age, residents of a long-term healthcare facility; 3) individuals between the ages of 18–49 with a medical condition; and 4) employees and residents in healthcare facilities at high risk of SARS-CoV-2 exposure and transmission [8, 9].

Immunity against SARS-CoV-2 induced with BNT126b2 vaccination provides a significant degree of protection. However, the duration of this protective immunity remains unknown. Many ongoing studies have focused on public concerns on the safety and efficacy of BNT126b2 over time [10]. Some studies have reported a significant antibody decrease three- and six months post-vaccination in individuals who completed the two-dose regimen [10–12]. Further, new strains of the SARS-CoV-2 virus could develop if it continues to replicate and be transmitted, and some may even become resistant to a vaccine [5].

The question of whether there is a need for a third dose remains open. Some countries have decided to apply a booster in severely immunocompromised individuals, but it is still unknown whether this would be necessary for the general public [13]. Therefore, this study aimed to measure SARS-CoV-2 spike 1–2 IgG antibodies in healthcare personnel vaccinated with the complete two-dose regimen of BNT126b2, and who received a third booster dose six months after the second dose.

## Material and methods

This was a prospective observational study that followed the STROBE guidelines [14]. It analyzed a subgroup of patients who completed the two-dose regimen of BNT126b2 in early 2021 in Monterrey, Nuevo León, Mexico. The study was approved by the local Institutional Review Board (Ref.:26022021-CN-1e-CI) and conducted per the Code of Ethics of the World Medical Association (Declaration of Helsinki) for experiments involving human subjects.

The inclusion criteria for the patients’ recruitment were: individuals of both genders between the age of 18 and 100 years old, who had accepted and signed an informed consent form, and had the intention of completing the BNT126b2 protocol. The patients were excluded if they did not complete the established vaccine regimen or received another SARS-CoV-2 vaccine.

The study subjects were introduced to the research protocol, specifying that it included a follow-up period of an entire year, the determination of SARS-CoV-2 specific IgG antibodies as well as the application of questionnaires. After agreeing, they signed a written informed consent form. Since this subgroup comprised healthcare personnel, in this country they were immunized against SARS-CoV-2 with BNT126b2 in early 2021. Since this protocol was approved after the application of the first BNT, twenty-one days after receiving this initial dose, the research team reached every participant to obtain a plasma sample for IgG antibody determination and the application of the first questionnaire. This questionnaire had the purpose of recording the patient’s medical history, their status of SARS-CoV-2 infections before and after immunization, and identifying adverse events following immunization (AEFI) [15, 16].

Twenty-one to 28 days after receiving the second dose, the participants were scheduled to provide the second sample for IgG antibody determination and complete a similar questionnaire to that previously applied. The third, fourth, and fifth IgG antibody samples were planned to be collected three, six, and twelve months after completing the two-dose regimen of BNT126b2. The questionnaires applied at the time of the blood collections had the aim of establishing the development of any suspicious or confirmed SARS-CoV-2 infections.

The plasma sample required 10 to 15 ml of blood obtained per venipuncture. These samples were placed in sample tubes with ethylenediaminetetraacetic acid (EDTA) as an anticoagulant and stored at -80°C. The equipment used by the laboratory personnel to analyze the samples was the LIAISON SARS-CoV-2 S1 / S2 IgG antibody detection kit (Italy), which uses magnetic beads coated with S1 & S2 antigens. To determine the amount of specific anti-S1 and anti-S2 IgG antibodies against SARS-CoV-2 in the plasma samples, the laboratory personnel used a chemiluminescence immunoassay (CLIA), with a sensitivity of 97.4% (95% CI, 86.8–99.5) and a specificity of 98.5% (95% CI, 97.5–99.2). The results were reported as follows: <12.0 AU / ml was considered negative, 12.0 to 15.0 AU / ml was indeterminate, and > 15 AU / ml was positive. The agreement in the detection of neutralizing antibodies is 94.4% [17, 18]. This kit is comparable to other commercial kits such as euroimmun and Roche and has been used in other studies by authors such as Fabrizio Bonelli et al., Maria Teresa Sandri et al., Riccardo Levi et al. [18–21].

Over the three-month follow-up, there was a decrease in the amount of specific anti-S1 and anti-S2 IgG antibodies against SARS-CoV-2. This finding raised concern among the healthcare personnel, leading them to seek a BNT126b2 booster on their own, thus infringing the original BNT regimen. Furthermore, six months after the two-dose BNT162b2 regimen, 168 participants received a third booster. Given the situation, two additional blood samples were collected from the participants: 1–7 days after the booster (once the research team was notified), and 21–28 days after the third booster.

The variables we analyzed were age, gender, medical history, including the confirmation of a SARS-CoV-2 diagnosis (either with a nasal swab or serologic tests before and after the BNT126b2 shots), and the development of AEFI any dose of BNT126b2. The analyzed biochemical variables were SARS-CoV-2 quantitative antibodies 21–28 days after the first BNT126b2 dose (S1), 21–28 days after the BNT126b2 second dose (S2), at the three-month follow-up after completing the two-dose BNT126b2 regimen (S3), and 1–7 days (S4) and 21–28 days after the BNT booster (S5).

The researchers reviewed the quality control parameters and the anonymization of the database. We presented results with descriptive statistics such as median, the interquartile range for quantitative variables, frequencies, and percentages for categorical variables. Group comparisons were established with Cochran’s Q test for categorical variables. The Mann Whitney U test was performed for the quantitative comparison of IgG antibodies between individuals with a positive vs. negative history of SARS-CoV-2 infection. Wilcoxon signed-range test was performed for IgG comparison between two time points of BNT126b2 dose. Additionally, we developed a mixed model in which the dependent variable was the antibody values. The reference group was the quantitative SARS-CoV-2 antibody total value 21–28 days after the first BNT126b2 dose (S1). Personal variation was constructed as a random effect, and every time antibodies were measured, subject gender and history of SARS-CoV-2 represented the statistical fixed effect. Completely missing random values were analyzed by complete case analysis. The statistical program used was SPSS, version 2. The analysis was two-tailed, and a p-value < 0.05 was considered statistically significant.

## Results

One hundred and sixty-eight (168) recruited participants received a three-dose BNT126b2 regimen, and 113 (67.3%) were female. Their mean (SD) age was 41 (10) years. The most common comorbidity was obesity (44, 26.2%), followed by dyslipidemia (15, 8.9%) and hypertension (14, 8.3%). Table 1 shows the participants´ medical histories as referred in the first questionnaire. The time (SD) between the first and second BNT126b2 vaccines was 31 (4) days, and between the second and third dose, it was 166 (12) days.

**Table 1:** Medical history.

Medical history (n = 168)	Frequency (%)
Obesity	44 (26.2)
Dyslipidemia	15 (8.9)
Hypertension	14 (8.3)
Diabetes	10 (6.0)
Prediabetes	6 (3.6)
Smoking	12 (7.1)
Pregnancy	1 (0.6)

Data are presented as frequencies and percentages.

### History of SARS-CoV-2

In terms of previous SARS-CoV-2 diagnoses, 95 (56.5%) of the participants reported never having the infection. Seventy-three (43.5%) participants referred a SARS-CoV-2 diagnosis before vaccination and 21–28 days after the third BNT booster. Before vaccination, there were 65 (38.7%) healthcare workers with one confirmed SARS-CoV-2 infection, and 7 (4.2%) that had been infected twice. There were 3 (1.8%) participants with a SARS-CoV-2 diagnosis between the first and second BNT doses, 1 (0.6%) between the second and third BNT doses, and none after the third dose. Table 2 shows the history of SARS-CoV-2 infection among participants, in frequencies and percentages.

**Table 2:** History of SARS-CoV-2 infection.

History of SARS-CoV-2 (n = 168)	Frequency (%)
**Positive history before vaccination**	
Once before vaccination	65 (38.7)
Twice before vaccination	7 (4.2)
**Positive history after initiating vaccination regimen**	
Between first and second BNT doses	3 (1.8)
Between second and third BNT doses	1 (0.6)
After third BNT dose	0 (0)

The table is divided into positive history before vaccination and after initiating the vaccination regimen.

### Antibody titers

The median (IQR) quantitative SARS-CoV-2 spike 1–2 IgG antibody titers (AU/ml) obtained in the healthcare personnel with no history of SARS-CoV-2 infection (n = 95) was 1350 (1224.0) after 21–28 days of BNT162b2 second dose, and in subjects with history of SARS-CoV-2 (n = 73), 2390 (2540.0), p = 0.002. Three months after two doses, antibody concentrations decreased 84.8% and 84.4%, in negative and positive SARS-CoV-2 participants, respectively. After 21–28 days of BNT162b2 third dose, AU/ml IgG titers increased to 2960 (2010.0) and 3090 (2080.0) in subjects with negative and positive history of SARS-CoV-2 respectively (p = 0.377). If we compare the IgG titters 21–28 days after second with 21–28 days after third BNT162b2 dose, in both groups (positive and negative history of SARS-CoV-2 infection), there is a statistical increase (p<0.001).

Table 3 presents the median (IQR) of the quantitative SARS-CoV-2 IgG antibody titers once the participants were divided into two groups according to their history of SARS-CoV-2 infection.

**Table 3:** Quantitative SARS-CoV-2 spike 1–2 IgG antibody titers against SARS-CoV-2 in participants with a positive or negative history of SARS-CoV-2 infection.

History of SARS-CoV-2 (n = 168) AU/ml	21–28 days after first BNT dose (S1) (IQR)	21–28 days after second BNT dose (S2) (IQR)	Three months after the second BNT dose (S3) (IQR)	1–7 days after the third BNT booster (S4) (IQR)	21–28 days after the third BNT booster (S5) (IQR)
**Negative (n = 95)**	103 (77.6)	1350 (1224.0)	205 (149.0)	379 (645.5)	2960 (2010.0)
**Positive (n = 73)**	1130 (4756.0)	2390 (2540.0)	377 (1144.7)	590 (1262.0)	3090 (2080.0)
**p-value**	<0.001	0.002	0.001	0.011	0.377

Data are presented as medians and interquartile ranges. The participants were divided into two groups, those with a negative and a positive history of SARS-CoV-2 infection.

**Fig 1:**
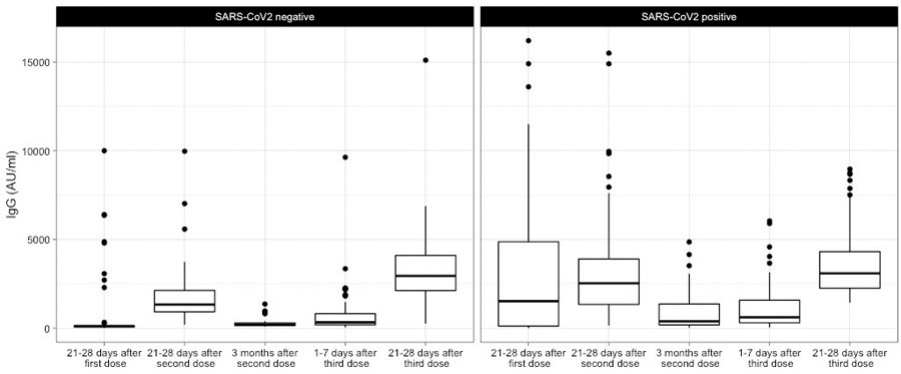
Boxplot scheme of the quantitative SARS-CoV-2 spike 1–2 IgG antibody titers according to SARS-CoV-2 history.

Fig 1 shows boxplot schemes of the quantitative SARS-CoV-2 spike 1–2 IgG antibody titers against SARS-CoV-2, to compare the results between groups with a negative or a positive SARS-CoV-2 history.

Quantitative SARS-CoV-2 spike 1–2 IgG titers after the first and second dose, three months, 1–7 days after the third dose, and 21–28 days following a BNT162b2 booster, dividing the population into two groups according to the SARS-CoV-2 history.

**Fig 2:**
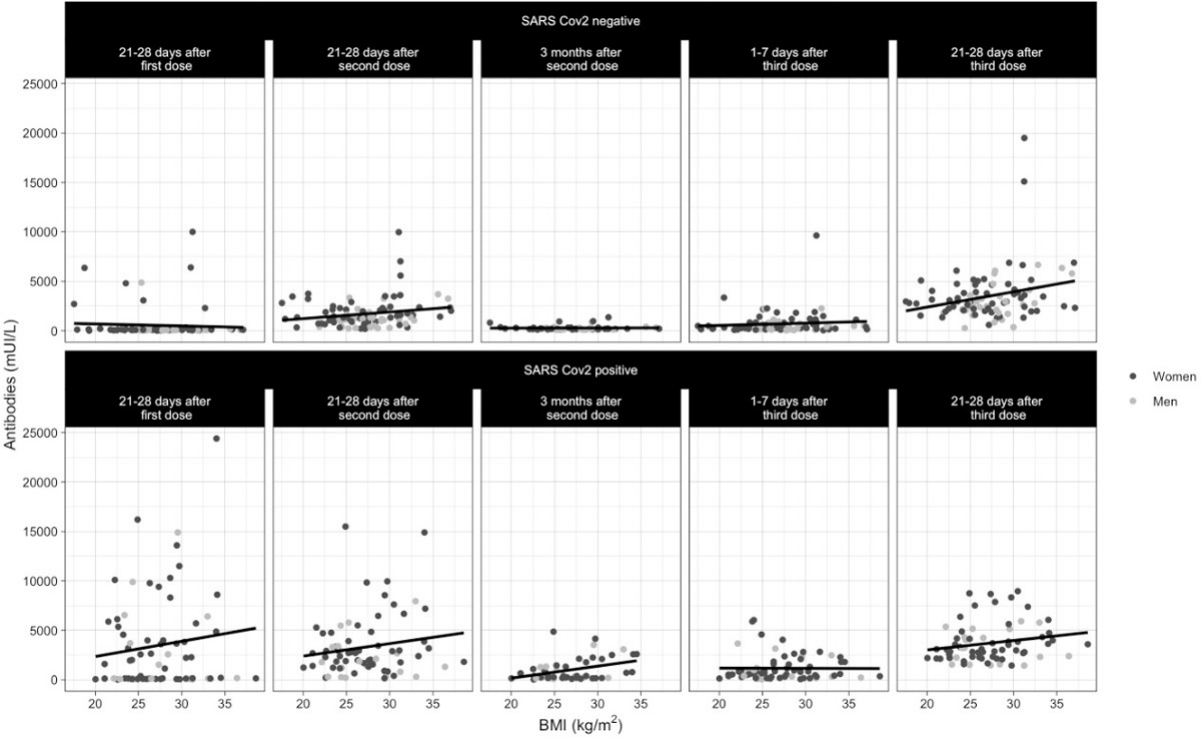
Effect of the third BNT126b2 dose, BMI, gender, and SARS-CoV-2 infection history.

We created a mixed model in which IgG levels 21–28 days after the BNT162b2 first dose were considered as the reference for comparisons. This model showed a difference in terms of patient gender since females had higher antibody titers (β = -652.3, p = 0.021). In addition, a higher BMI was associated with an increase in antibody levels (β = 74.5 p = 0.02). There was a positive effect in subjects with a positive SARS-CoV-2 history (β = 1147.9, p< 0.001), and the antibody levels changed over time (p<0.01). When we compared the antibody titers 21–28 days after the second and third doses, there was a greater increase after the latter dose (β = 591.5, p = 0.004 and β = 1782.3, p< 0.001, respectively). Table 4 and Fig 2.

**Table 4:** Mixed model of the SARS-CoV-2 spike 1–2 IgG antibody titers over time.

Variable	Estimate (β)	Std. Error	95% CI	p-value
Intercept	-506.12	887.16	381.04,-1393.28	0.569
Gender	-652.3	279.5	-1,205, -100	0.021
BMI	74.5	31.6	12, 137	0.02
21–28 days after second dose	591.5	205.7	187, 996	0.004
3 months after second dose	-1155.2	237	-1,620, -691	<0.001
1–7 days after third dose	-910.8	205.7	-1,315, -507	<0.001
21–28 days after third dose	1782.3	205.7	1,378, 2,186	<0.001
SARS-CoV-2	1147.9	263	629, 1,667	<0.001

Mixed model in which the dependent variable was the quantitative SARS-CoV-2 spike 1–2 IgG antibody titers. Antibodies determined 21–28 days after the first BNT126b2 were the reference group. AIC: 13595.

Effect of each BNT126b2 dose, showing an increase in quantitative SARS-CoV-2 spike 1–2 IgG antibodies. Variables such as a positive SARS-Cov2 infection history, female sex, and a high BMI led to a greater increase in antibody titers.

### Adverse events following immunization (AEFI)

AEFI were reported after the first, second, and third BNT doses; the lowest total number of events were reported after the third BNT162b2 dose (n = 140, 83.0%; n = 133, 79.1%; and n = 97, 57.7%, respectively) (p<0.001). After the first and second BNT doses, AEFI developed most frequently within the first four hours after application (n = 116, 82.8%; and n = 59, 44.4%, respectively), whereas after the third vaccine, AEFI were reported 5 to 24 hours later (n = 56, 57.7%). Their severity was referred to as very mild after the first and second BNT doses (n = 95, 67.8%; and n = 57, 42.8%, respectively), and mild after the third BNT booster (n = 35, 36.0%).

The most common AEFI resulting from the three administered vaccines were pain at the injection site (n = 131, 93.6%; n = 119, 89.5%; and n = 82, 84.0%, respectively), followed by headache (n = 51, 36.4%; n = 58, 43.6%; and 43, 44.3%, respectively), and fatigue (n = 38, 27.1%; n = 54, 32.1%; and n = 45, 46.4%, respectively). Specifically, when comparing the AEFI after each dose, fatigue, myalgias, arthralgias, fever, and adenopathy were proportionally more frequent following the third dose, than with the two-dose regimen (p<0.05). Table 5 shows the AEFI reported by the participants in frequencies and percentages, and Table 6 shows the time of their presentation and their severity.

**Table 5:** Adverse events following immunization after each administered BNT162b2 dose.

AEFI (n = 168)	First dose (%)	Second dose (%)	Third dose (%)	p-value
Present	140 (83.3)	133 (79.1)	97 (57.7)	< 0.001
Symptoms				
**Pain at the injection site**	131 (93.6)	119 (89.5)	82 (84.5)	0.204
**Headache**	51 (36.4)	58 (43.6)	43 (44.3)	0.459
**Fatigue**	38 (27.1)	54 (40.6)	45 (46.4)	0.030
**Myalgia**	12 (8.6)	38 (22.6)	27 (27.8)	0.001
**Local edema or erythema**	11 (6.5)	20 (15.0)	15 (15.5)	0.421
**Arthralgia**	8 (5.7)	37 (22.0)	26 (26.8)	0.001
**Fever**	4 (2.9)	4 (3.0)	11 (11.3)	0.003
**Insomnia**	5 (3.0)	3 (2.3)	5 (5.2)	0.561
**Palpitations**	4 (2.4)	7 (5.3)	7 (7.2)	0.641
**Chest pain or oppression**	5 (3.0)	6 (4.6)	5 (5.2)	0.846
**Nasal congestion**	4 (2.9)	17 (12.8)	8 (8.2)	0.047
**Adenopathy**	1 (0.7)	3 (2.3)	14 (14.4)	0.001
**Hypotension**	1 (0.7)	2 (1.5)	2 (2.1)	0.368
**Pruritus**	1 (0.7)	4 (3.0)	3 (3.1)	0.247
**Ocular pain**	2 (1.7)	0	0	0.368
**Nausea**	4 (2.9)	11 (8.3)	6 (6.2)	0.035
**Diarrhea**	0	5 (3.8)	4 (4.1)	0.135

Data are presented as frequencies and percentages; reported AEFI, the most frequent, time of appearance, and severity. Cochran’s Q test was performed for comparisons between the AEFI after each dose. A p-value ≤ 0.05 was considered statistically significant.

**Table 6:** Time of appearance and severity of AEFI after each BNT162b2 dose.

Time of appearance of adverse events after immunization			
	First dose	Second dose	Third dose
**First four hours after vaccination**	116 (82.8)	59 (44.4)	19 (19.6)
**Five to 24 hours after vaccination**	6 (4.3)	48 (36.1)	56 (57.7)
**Two to three days after vaccination**	18 (12.9)	25 (18.8)	21 (21.6)
**4–7 days after vaccination**	0	1 (0.8)	0
**7–10 days after vaccination**	0	0	0
**> 10 days after vaccination**	0	0	1 (1.0)
**Severity**			
**Very mild**	95 (67.8)	57 (42.8)	35 (36.0)
**Mild**	18 (12.8)	47 (35.3)	36 (37.1)
**Moderate**	7 (5.0)	27 (20.3)	24 (24.7)
**Severe**	0	2 (1.5)	0

Data are presented as frequencies and percentages.

## Discussion

The purpose of this study was to analyze the quantitative SARS-CoV-2 spike 1–2 IgG antibody titers over six months after completing the established BNT126b2 vaccination regimen, and 21–28 days following a third BNT126b2 dose applied six months after the second dose. Three months after the second dose, antibodies decreased in comparison with the titers determined 21–28 days after the second dose. In addition, in the sample collected 1–7 days after the third dose—equivalent to the sample after six months of the complete regimen -, the antibody titers were lower than those reported 21–28 days after the second dose.

Previous studies have demonstrated a decrease in the quantitative SARS-CoV-2 spike 1–2 IgG antibody titers after completing the BNT126b2 protocol, regardless of the patients´ SARS-CoV-2 history. Alejo Erice et al. reported a 58% decrease in the quantitative SARS-CoV-2 spike 1–2 IgG antibody titers after three months [22].

In addition, the study conducted by Julien Favresse et al. referred a decrease in antibody titers 56 to 90 days after the second BNT162b2 vaccine, in participants that were seronegative and seropositive at the beginning of the study [11]. Our study was concordant with these previous investigations in terms of the decrease in the quantitative SARS-CoV-2 spike 1–2 IgG antibody titers observed in the third sample. Three months after completion of the BNT162b2 protocol, antibodies decreased 84.8% and 84.4% in negative and positive SARS-CoV-2 infection participants, respectively.

Twenty-one to 28 days after the administration of the third BNT162b2 booster, the quantitative SARS-CoV-2 spike 1–2 IgG antibody titers increased drastically. Participants with a negative SARS-CoV-2 infection increased their antibody titers over six-fold in comparison with the previous results obtained 1–7 days after the third dose; in positive SARS-CoV-2 infection cases, titers increased four-fold. According to our mixed model, when we compared the estimates from days 21–28 after the second dose and 21–28 after the third dose, there was a greater increase in antibody levels after the third dose.

In a study in which heart transplant recipients were vaccinated with a third BNT162b2 dose, Yael Peled, et al. reported a three-fold increase in anti-receptor binding domain antibodies, despite immunosuppressive therapy [23]. Additionally, according to Yinon M Bar-On et al., after a third BNT162b2 booster dose was administered to an Israeli group of individuals over 60 years of age, the rates of confirmed SARS-CoV-2 infection and severe illness decreased in comparison with a control group immunized with the original BNT162b2 two-dose regimen [24]. Given these situations and our findings, a regimen of three homologous BNT162b2 doses regimen in which the third dose is administered six months after the second vaccine, provides a beneficial effect in terms of a positive humoral immune response against SARS-CoV-2 infection.

During the six months before the third dose, we noticed an increase in the quantitative SARS-CoV-2 spike 1–2 IgG antibody titers among participants with a positive history of SARS-CoV-2, in comparison with those with a negative history. Interestingly, in the results obtained 21–28 days after the third booster dose, the difference between the participants with a positive or negative SARS-CoV-2 history was not as perceptible as with the first two vaccines. This suggests that after the third dose, participants with a negative SARS-CoV-2 history have the same level of humoral immunity as those previously exposed to a SARS-CoV-2 infection.

Our mixed model showed interesting results in terms of patient gender and BMI. A study by Pellini et al. analyzed the differences between genders in the quantitative SARS-CoV-2 spike 1–2 IgG antibody titers. Seven days following the second BNT162b2 dose, the researchers found that females developed a superior humoral response. Although our study had a longer follow-up period as well as the analysis of plasma after a third dose, the trend was similar, since women developed higher antibody titers than men following immunization [25].

In terms of the BMI, we found an opposite result to that of Pellini et al. We found that a higher BMI was associated with an increase in antibody levels. Since the prevalence of obesity in Mexico and Italy is different, we believe that there might be a relation between a greater increase in antibodies and extreme values of BMI, either a high or low BMI. Models based on cubic relations should be further explored [25].

The main concern with the application of the third dose was the lack of information on the development of possible AEFI. Yael Peled et al. reported that 67% of participants who received a third BNT162b2 booster reported at least one AEFI of mild severity. The most common AEFI were pain at the injection site, fatigue, and headache, but no hospitalizations or emergency room (ER) visits (23) were necessary. Our study also found that the AEFI associated with the third dose were mild, and the most frequent were pain at the injection site, fatigue, and headache, with no associated severe complications such as hospitalization or ER visits. In comparison with the other doses, the total number of AEFI caused by the third dose was minimal; they decreased 30.7% and 27.0% in comparison with those reported after the first and second BNT162b2 shots, respectively.

To the best of our knowledge, no previous studies have shown the quantitative SARS-CoV-2 spike 1–2 IgG antibody titer response to a third BNT162b2 booster in non-immunocompromised patients. We believe that this study may contribute to further the healthcare community´s knowledge since we observed a positive humoral immune response to the third BNT162b2 dose in our healthcare personnel. This can lead to better protection among healthcare personnel and in groups that are vulnerable to severe illness by SARS-CoV-2.

Approval of the third BNT162b2 dose and its proven efficacy could also promote better control of the SARS-CoV-2 pandemic, providing greater immunogenicity among the vulnerable population. Consequently, the number of confirmed cases and hospitalizations in Mexico could decrease.

A limitation to our study is the fact that the recruited group did not have a baseline sample obtained before the first BNT162b2 dose because our protocol was approved after the health personnel had received the first dose. However, we believe that this study is significant since our population was not immunocompromised. Another limitation is that the analysis of the third dose’s adverse events after immunization was only conducted in the short term (21–28 days after the booster dose). We will continue following this cohort to determine any long-term AEFI related to the vaccine since we have only reported the short-term AEFI in this study. Another limitation of this study was that the assay used to measure IgG did not discriminate between immunization or direct exposure to SARS-CoV-2. Finally, although the positive concordance of quantitative SARS-CoV-2 spike 1–2 IgG antibody titers with neutralizing antibodies has been reported to be 94.4%, it would be of interest to measure the latter in our population [17, 18].

## Conclusion

The third BNT162b2 booster dose has proven to increase the quantitative SARS-CoV-2 spike 1–2 IgG antibody titers with no severe short-term AEFI. Despite its positive response, we must continue studying this cohort to establish whether the antibody titers persist and SARS-CoV-2 infections and their consequent severe illness decrease.

BNT162b2 Pfizer/BioNTech is one of the most studied vaccines due to its efficacy and worldwide application. After approval of its third dose by the CDC and FDA, further research on BNT162b2 and its new vaccination protocol will provide critical information to the medical field, hence contributing to better control of the pandemic. Our cohort study may be considered significant since no other studies quantitatively measuring SARS-CoV-2 spike 1–2 IgG antibody titers in healthcare personnel had been found when this article was written.

We will continue pursuing our study to report further findings that may provide more relevant information that could contribute to the control of the SARS-CoV-2 pandemic.

## Supporting information

S1 Data (XLSX)

## References

[1] World Health Organization. WHO Director-General’s opening remarks at the media briefing on COVID-19–11 March 2020 [Internet]. 2020 [cited 2021 Oct 5]. Available from: https://www.who.int/director-general/speeches/detail/who-director-general-s-opening-remarks-at-the-media-briefing-on-covid-19—11-march-2020

[2] CEPAL N OPS. The prolongation of the health crisis and its impact on health, the economy and social development. 2021 Oct 14 [cited 2022 Jan 3]; Available from: https://repositorio.cepal.org/handle/11362/47302

[3] Jagtap S, R K, Valloly P, Sharma R, Maurya S, Gaigore A, et al. Evaluation of spike protein antigens for SARS-CoV-2 serology. J Virol Methods. 2021 Jun 16;296:114222. doi: 10.1016/j.jviromet.2021.114222 34197839 PMC8239204

[4] Sadarangani M, Marchant A, Kollmann TR. Immunological mechanisms of vaccine-induced protection against COVID-19 in humans. Nat Rev Immunol. 2021 Jul 1;21(8):475–84. doi: 10.1038/s41577-021-00578-z 34211186 PMC8246128

[5] Badiani AA, Patel JA, Ziolkowski K, Nielsen FBH. Pfizer: The miracle vaccine for COVID-19? Public Health in Practice. 2020 Nov;1:100061. doi: 10.1016/j.puhip.2020.100061 34173584 PMC7754880

[6] Lamb YN. BNT162b2 mRNA COVID-19 Vaccine: First Approval. Drugs. 2021 Mar;81(4):495–501. doi: 10.1007/s40265-021-01480-7 33683637 PMC7938284

[7] Food and Drug Administration. FDA Takes Key Action in Fight Against COVID-19 By Issuing Emergency Use Authorization for First COVID-19 Vaccine [Internet]. FDA. FDA; 2020 [cited 2021 Oct 8]. Available from: https://www.fda.gov/news-events/press-announcements/fda-takes-key-action-fight-against-covid-19-issuing-emergency-use-authorization-first-covid-19

[8] CDC. COVID-19 Vaccination [Internet]. Centers for Disease Control and Prevention. 2020 [cited 2021 Oct 15]. Available from: https://www.cdc.gov/coronavirus/2019-ncov/vaccines/booster-shot.html

[9] Commissioner O of the. FDA Authorizes Booster Dose of Pfizer-BioNTech COVID-19 Vaccine for Certain Populations [Internet]. FDA. FDA; 2021 [cited 2021 Oct 15]. Available from: https://www.fda.gov/news-events/press-announcements/fda-authorizes-booster-dose-pfizer-biontech-covid-19-vaccine-certain-populations

[10] Kontopoulou K, Nakas C, Ntenti C, Katsioulis C, Goulas A, Papazisis G. Antibody Titers 3-Months Post-Vaccination with the Pfizer/Biontech Vaccine in Greece [Internet]. Rochester, NY: Social Science Research Network; 2021 Aug [cited 2021 Oct 9]. Report No.: ID 3899094. Available from: https://papers.ssrn.com/abstract=3899094

[11] Favresse J, Bayart J-L, Mullier F, Elsen M, Eucher C, Van Eeckhoudt S, et al. Antibody titres decline 3-month post-vaccination with BNT162b2. Emerg Microbes Infect. 2021 Dec;10(1):1495–8. doi: 10.1080/22221751.2021.1953403 34232116 PMC8300930

[12] Salvagno GL, Henry B, Pighi L, De Nitto S, Lippi G. Total Anti-SARS-CoV-2 Antibodies Measured 6 Months After Pfizer-BioNTech COVID-19 Vaccination in Healthcare Workers. SSRN Journal [Internet]. 2021 [cited 2021 Oct 9]; Available from: https://www.ssrn.com/abstract=3915349 10.5937/jomb0-33999 PMC9010038 35510202

[13] Tré-Hardy M, Cupaiolo R, Wilmet A, Antoine-Moussiaux T, Della Vecchia A, Horeanga A, et al. Six-month interim analysis of ongoing immunogenicity surveillance of the mRNA-1273 vaccine in healthcare workers: A third dose is expected. Journal of Infection [Internet]. 2021 Aug 23 [cited 2021 Oct 9]; Available from: https://www.sciencedirect.com/science/article/pii/S0163445321004333

[14] Cuschieri S. The STROBE guidelines. Saudi J Anaesth. 2019 Apr;13(Suppl 1):S31–4. doi: 10.4103/sja.SJA_543_18 30930717 PMC6398292

[15] Chen M, Yuan Y, Zhou Y, Deng Z, Zhao J, Feng F, et al. Safety of SARS-CoV-2 vaccines: a systematic review and meta-analysis of randomized controlled trials. Infect Dis Poverty. 2021 Jul 5;10(1):94. doi: 10.1186/s40249-021-00878-5 34225791 PMC8256217

[16] Fan Y-J, Chan K-H, Hung IF-N. Safety and Efficacy of COVID-19 Vaccines: A Systematic Review and Meta-Analysis of Different Vaccines at Phase 3. Vaccines (Basel). 2021 Sep 4;9(9):989. doi: 10.3390/vaccines9090989 34579226 PMC8473448

[17] DiaSorin’s LIAISON® SARS-CoV-2 Diagnostic Solutions [Internet]. DiaSorin. [cited 2022 Jan 3]. Available from: https://www.diasorin.com/en/immunodiagnostic-solutions/clinical-areas/infectious-diseases/covid-19

[18] Bonelli F, Sarasini A, Zierold C, Calleri M, Bonetti A, Vismara C, et al. Clinical and Analytical Performance of an Automated Serological Test That Identifies S1/S2-Neutralizing IgG in COVID-19 Patients Semiquantitatively. J Clin Microbiol. 2020 Aug 24;58(9):e01224–20. doi: 10.1128/JCM.01224-20 32580948 PMC7448652

[19] Sandri MT, Azzolini E, Torri V, Carloni S, Pozzi C, Salvatici M, et al. SARS-CoV-2 serology in 4000 health care and administrative staff across seven sites in Lombardy, Italy [Internet]. 2020 Dec [cited 2022 Jan 3] p. 2020.05.24.20111245. Available from: https://www.medrxiv.org/content/10.1101/2020.05.24.20111245v2 10.1038/s41598-021-91773-4 PMC8192543 34112899

[20] Levi R, Azzolini E, Pozzi C, Ubaldi L, Lagioia M, Mantovani A, et al. One dose of SARS-CoV-2 vaccine exponentially increases antibodies in individuals who have recovered from symptomatic COVID-19. J Clin Invest. 2021 Jun 15;131(12):149154. doi: 10.1172/JCI149154 33956667 PMC8203458

[21] Levi R, Ubaldi L, Pozzi C, Angelotti G, Sandri MT, Azzolini E, et al. The antibody response to SARS-CoV-2 infection persists over at least 8 months in symptomatic patients. Commun Med. 2021 Sep 17;1(1):1–9. doi: 10.1038/s43856-021-00032-0 35072166 PMC8767777

[22] Erice A, Varillas-Delgado D, Caballero C. Decline of antibody titres 3 months after two doses of BNT162b2 in non-immunocompromised adults. Clinical Microbiology and Infection [Internet]. 2021 Sep 9 [cited 2021 Oct 14]; Available from: https://www.sciencedirect.com/science/article/pii/S1198743X21004857 doi: 10.1016/j.cmi.2021.08.023 34508885 PMC8426320

[23] Peled Y, Ram E, Lavee J, Segev A, Matezki S, Wieder-Finesod A, et al. Third dose of the BNT162b2 vaccine in heart transplant recipients: Immunogenicity and clinical experience. J Heart Lung Transplant [Internet]. 2021 Aug 28 [cited 2021 Oct 15]; Available from: https://www.ncbi.nlm.nih.gov/pmc/articles/PMC8397500/ doi: 10.1016/j.healun.2021.08.010 34565682 PMC8397500

[24] Bar-On YM, Goldberg Y, Mandel M, Bodenheimer O, Freedman L, Kalkstein N, et al. Protection of BNT162b2 Vaccine Booster against Covid-19 in Israel. N Engl J Med. 2021 Sep 15;NEJMoa2114255. doi: 10.1056/NEJMoa2114255 34525275 PMC8461568

[25] Pellini R, Venuti A, Pimpinelli F, Abril E, Blandino G, Campo F, et al. Initial observations on age, gender, BMI and hypertension in antibody responses to SARS-CoV-2 BNT162b2 vaccine. EClinicalMedicine. 2021 Jun;36:100928. doi: 10.1016/j.eclinm.2021.100928 34109307 PMC8177433

